# Fatal inflammation: understanding meningitis mortality in the United States (1999–2020)

**DOI:** 10.1186/s12883-026-05060-8

**Published:** 2026-06-13

**Authors:** Ali Shan Hafeez, Muhammad Faizan, Shaikh Muhammad Daniyal, Asad Zaman, Abdul Rafae Faisal, Abdullah, FNU Laiba, Rick Maity, Muhammad Abdullah Humayun, Pramod Singh, Mohammad Maaz Alam, Arkadeep Dhali

**Affiliations:** 1 CMH Multan Institute of Medical Sciences, Multan, Pakistan; 2 Hamad Medical Corporation, Doha, Qatar; 3 Dow University of Health Sciences, Karachi, Pakistan; 4 Rawalpindi Medical University, Rawalpindi, Pakistan; 5 Akhtar Saeed Medical and Dental College, Lahore, Pakistan; 6 School of Medical Science and Technology, Indian Institute of Technology Kharagpur, Kharagpur, India; 7 Mayo Clinic, Scottsdale, Arizona USA; 8 Barhabise Primary Health Care Centre, Sindhupalchowk, Nepal; 9 PNS Shifa Hospital, Karachi, Pakistan; 10 Sheffield Teaching Hospitals NHS Foundation Trust, Sheffield, UK; 11 School of Medicine, Dentistry and Biomedical Sciences, Queen’s University Belfast, Belfast, BT9 7BL United Kingdom

**Keywords:** Meningitis, Mortality rate, Epidemiology, Trend, CDC, Public health

## Abstract

**Background:**

Meningitis, an inflammatory condition that affects the meninges, has reported an increase in cases between 2006 and 2016. While in well-resourced settings, the incidence of acute bacterial meningitis has declined to below 0.5–1.5 cases per 100,000 population. Overall, late excess mortality following meningitis is highest within the first two years after discharge, particularly affecting patients aged 30–60 years. This research seeks to analyse these trends and investigate potential disparities in mortality rates based on demographic variables between 1999 and 2020.

By quantifying high-risk populations and regional differences, the findings provide evidence to guide targeted public health interventions and resource allocation.

**Methods:**

This retrospective observational study used CDC WONDER data (1999–2020) to analyse mortality trends for individuals aged 1–85 + years. Deaths related to meningitis were identified using ICD-10 codes A17.0, A32.1, A39.0, A87.0, A87.1, A87.2, A87.8, A87.9, B00.3, B01.0, B02.1, B05.1, B26.1, B37.5, B38.4, G00.0, G00.1, G00.2, G00.3, G00.8, G00.9, G03.0, G03.1, G03.2, G03.8, G03.9. Crude mortality rates and age-adjusted mortality rates (AAMRs) were calculated. Temporal trends and significant changes in mortality trajectories were assessed using Joinpoint regression analysis, which estimated annual percentage changes (APCs) and identified statistically significant inflection points. Mortality patterns were further stratified by sex, race or ethnicity, census region, state, and urban–rural classification.

**Results:**

Between 1999 and 2020, meningitis-related AAMRs in the United States declined from 8.43 to 3.88 per 1,000,000 population, with the steepest decrease between 1999 and 2012 before stabilising thereafter. Males consistently showed higher mortality (AAMR 5.79) compared to females (AAMR 4.53), though both declined substantially over the study period. Non-Hispanic (NH) Black individuals exhibited the highest AAMRs (8.59), followed by Hispanics (4.81), NH Whites (4.64), and NH Asians or Pacific Islanders (3.58). Geographically, the West (5.35) and South (5.33) had the greatest burden, while the Northeast (4.69) and Midwest (4.81) reported lower rates. State-level variation ranged from 8.85 per 1,000,000 in the District of Columbia to 3.52 per 1,000,000 in Delaware, with higher mortality in non-metropolitan areas than metropolitan ones.

**Conclusion:**

This study provides a comprehensive analysis of meningitis-related mortality trends in the United States from 1999 to 2020, highlighting significant declines in AAMRs across demographic groups, regions, and urbanisation levels. Despite the overall decline, the stabilisation of AAMRs in the last decade suggests a plateau in progress, necessitating further investigation into the underlying factors. Thus, targeted interventions to enhance the prevention, control, and management of risk factors might be required to achieve lasting change.

**Supplementary Information:**

The online version contains supplementary material available at 10.1186/s12883-026-05060-8.

## Introduction

Meningitis is an inflammatory condition that affects the meninges, which surround and safeguard the brain and spinal cord [1]. The disease can arise from bacterial, viral, fungal, or parasitic infections, with bacterial meningitis representing the most severe form due to its rapid progression and high fatality rates. Globally, the reported cases of bacterial meningitis at surveillance sites increased between 2006 and 2016, with incidence strongly associated with poverty as indicated by the Socio-Demographic Index (SDI), whereas, in well-resourced settings, the incidence of acute bacterial meningitis has declined to below 0.5–1.5 cases per 100,000 population [2]. In the United States, substantial progress has been made in reducing meningitis incidence and mortality through vaccination programs targeting major pathogens [3]. Overall, late excess mortality following meningitis is highest within the first two years after discharge, with a five-year cumulative rate particularly elevated in patients aged 30–60 years and those with severe initial presentations .Social distancing measures implemented to mitigate the spread of SARS-CoV-2 during the COVID-19 pandemic were also projected to result in a 20–30% reduction in meningitis incidence [3, 4]. Furthermore, disparities in disease burden and outcomes persist across demographic groups, including differences related to age, sex, race, and geographic location within the United States. Moreover, existing literature has not systematically examined disparities in meningitis-related mortality across urban and rural populations, nor has it comprehensively quantified the magnitude of racial and geographic differences in mortality burden across the United States. Comprehensive analyses examining long-term national mortality trends across multiple demographic strata remain limited. In particular, there is insufficient understanding of how meningitis-related mortality patterns have evolved over time in the United States. This research seeks to analyse these trends via the Centers for Disease Control and Prevention (CDC) Wide-ranging ONline Data for Epidemiologic Research (WONDER) Database between 1999 and 2020. We also aim to investigate potential disparities in mortality rates based on demographic variables such as sex, race, and region, and identify possible factors underlying these differences. Identifying vulnerable populations and regions is crucial for effectively implementing targeted interventions to prevent disease progression.

## Methods

### Study design

Mortality data were sourced from the CDC WONDER Database [5]. CDC WONDER is an extensive database that encompasses death certificate information from all 50 U.S. states and the District of Columbia. Data on meningitis-related deaths from 1999 to 2020 were analysed, comprising individuals aged < 1 to 85 + years, using the following International Classification of Diseases, Tenth Revision (ICD-10) codes: A17.0, A32.1, A39.0, A87.0-A87.2, A87.8, A87.9, B00.3, B01.0, B02.1, B05.1, B26.1, B37.5, B38.4, G00.0-G00.3, G00.8-G00.9, G03.0-G03.2, and G03.8-G03.9. Multiple cause-of-death data were used to identify instances in which meningitis was recorded as either a contributory or underlying cause of death.

Since this study made use of de-identified, publicly available datasets from a government database, institutional review board approval was not required. This study adheres to the reporting standards outlined in the Strengthening the Reporting of Observational Studies in Epidemiology (STROBE) guidelines [6]. Data were restricted to 1999–2020 because mortality counts for subsequent years were unstable and frequently suppressed within CDC WONDER due to very small death counts, which produces unreliable rate estimates and artificial trend distortions. Consistent with CDC reporting guidance, years with sparse mortality data were excluded to preserve statistical validity of Joinpoint regression and rate calculations.

### Data extraction

Data were categorised based on population, year, sex, race or ethnicity, urban-rural classification, census region, and state. Race and ethnicity classifications comprised Hispanic or Latino, Non-Hispanic (NH) Black or African American, NH White, NH Asian or Pacific Islander, and NH American Indian or Alaska Native.

Census regions were classified as Northeast, Midwest, South, and West. As per the 2013 U.S.

Census Bureau definitions, regions with a population of 50,000 or more were categorised as urban, whereas those with a population under 50,000 were classified as rural [7]. Non-Hispanic American Indian or Alaska Native populations were excluded from race-stratified trend analyses because CDC WONDER frequently suppresses mortality counts in this group due to small numbers, yielding statistically unreliable rate estimates. All mortality data were extracted directly from CDC WONDER. Raw query parameters and outputs are provided in the supplementary material to ensure reproducibility.

### Statistical analysis

Mortality rates were expressed per 1,000,000 population. The age-adjusted mortality rate (AAMR) was derived from age-specific mortality rates based on the U.S. 2000 standard population, enabling comparisons across different groups and time periods. AAMRs were analysed by sex, race or ethnicity, region, state, and urban or rural areas. The Joinpoint Regression Program (version 5.0.2) was used to compute the annual percentage changes (APCs) and average APC in AAMR, along with 95% confidence intervals (CIs). The program identifies trends to establish the simplest joinpoint model, starting with the minimum number of joinpoints. It then checks to see if the slope of mortality changes significantly by using a two-tailed t-test, considering a p-value of less than 0.05 statistically significant. Formal interaction testing between race and age was not possible due to aggregate-level data constraints.

## Results

### Overall trend

**Fig. 1:**
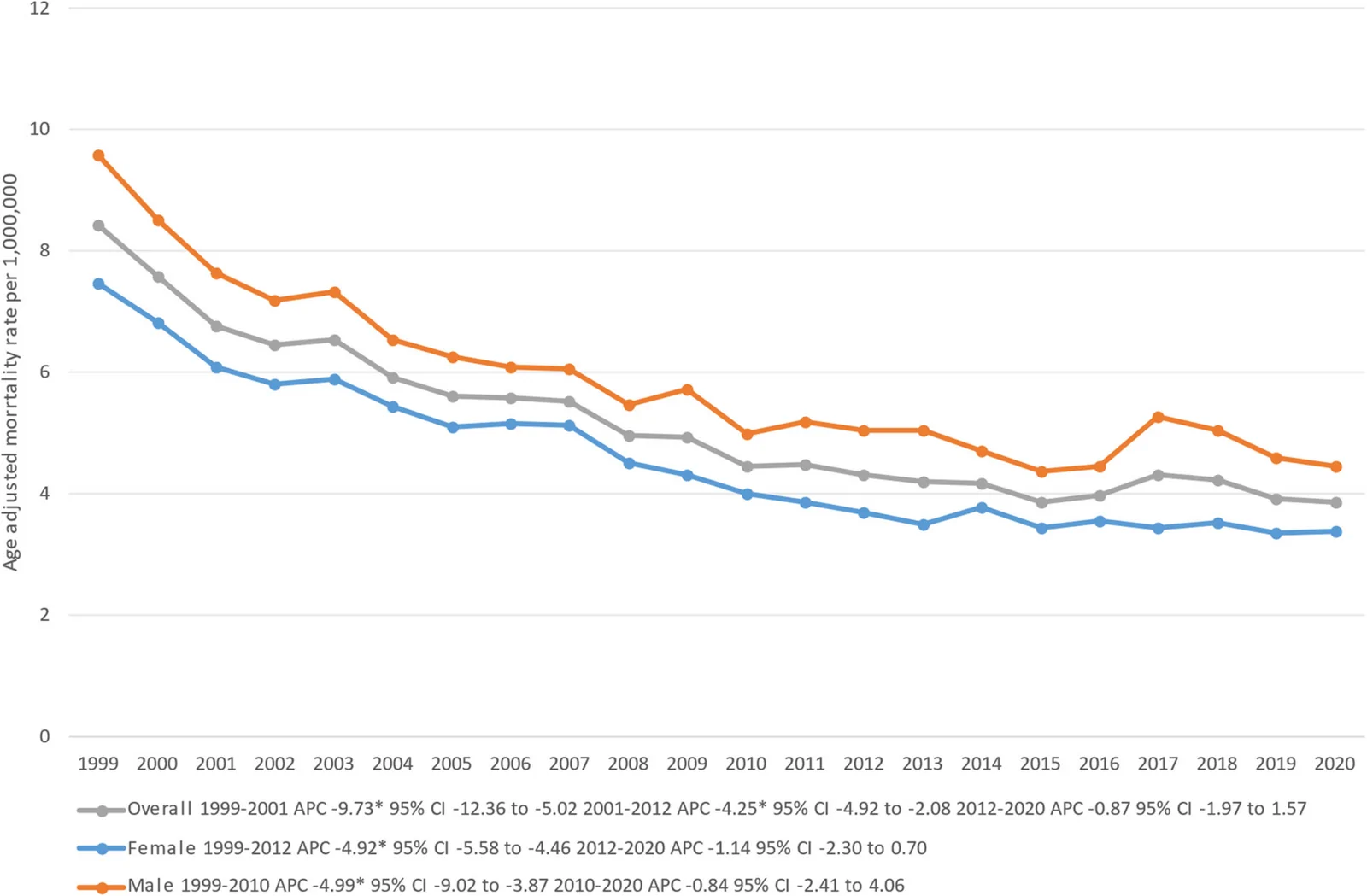
Overall and sex-stratified meningitis-related age adjusted mortality rates per 1,000,000 population in the United States, 1999 to 2020. * Annual percentage change is significantly different from zero at α = 0.05. AAMR = age-adjusted mortality rate; APC = annual percentage change; CI = confidence interval

With an overall value of 5.1 (95% CI: 5.05 to 5.15), the AAMR demonstrated a notable declining trend throughout the study period. Between 1999 and 2001, the AAMR significantly decreased from 8.43 to 6.78, with an APC of − 9.74* (95% CI: -12.37 to -5.02). This was followed by a continued significant decline until 2012, reaching 4.31 (APC: -4.25*; 95% CI: -4.92 to -2.08). After 2012, the AAMR remained relatively stable till 2020, with an APC of -0.88 (95% CI: -1.97 to 1.58) (Supplementary Tables 1 and 2) (Fig. 1).

### Sex distribution

Males consistently exhibited higher mortality rates (mean rate: 5.79, 95% CI: 5.71 to 5.87) than females (mean rate: 4.53, 95% CI: 4.46 to 4.6). The AAMR for males decreased significantly from 9.59 in 1999 to 4.45 in 2020. This decline was most significant during the initial period from 1999 to 2010, with an APC of − 4.99* (95% CI: −9.03 to − 3.87). Similarly, the AAMR for females due to meningitis dropped from 7.47 in 1999 to 3.38 in 2020. The most substantial decline in female mortality rates occurred from 1999 to 2012, with an APC of − 4.92* (95% CI: −5.59 to − 4.46). Following 2010 for males and 2012 for females, the AAMR remained relatively stable until 2020 (Supplementary Tables 1,2 and 3) (Fig. 1).

### Trends by race or ethnicity

Among the racial and ethnic groups, NH Black individuals displayed the highest overall AAMR of 8.59 (95% CI: 8.38 to 8.79), followed by Hispanics/Latinos 4.81 (95% CI: 4.64 to 4.97), NH White 4.64 (95% CI: 4.58 to 4.7), and NH Asian or Pacific Islander which had an AAMR of 3.58 (95% CI: 3.36 to 3.79). All ethnicities demonstrated an initial significant decrease in mortality rates. NH Black individuals experienced a notable decline from 1999 to 2001 (APC: -14.21*, 95% CI: -17.03 to -8.24). Both NH Black and NH White populations showed continued significant decreases until 2012 (APC: -7.23*, 95% CI: -8.11 to -2.93) and (APC: -3.93*, 95% CI: -6.63 to -3.26) respectively, followed by a stable period till 2020.

**Fig. 2:**
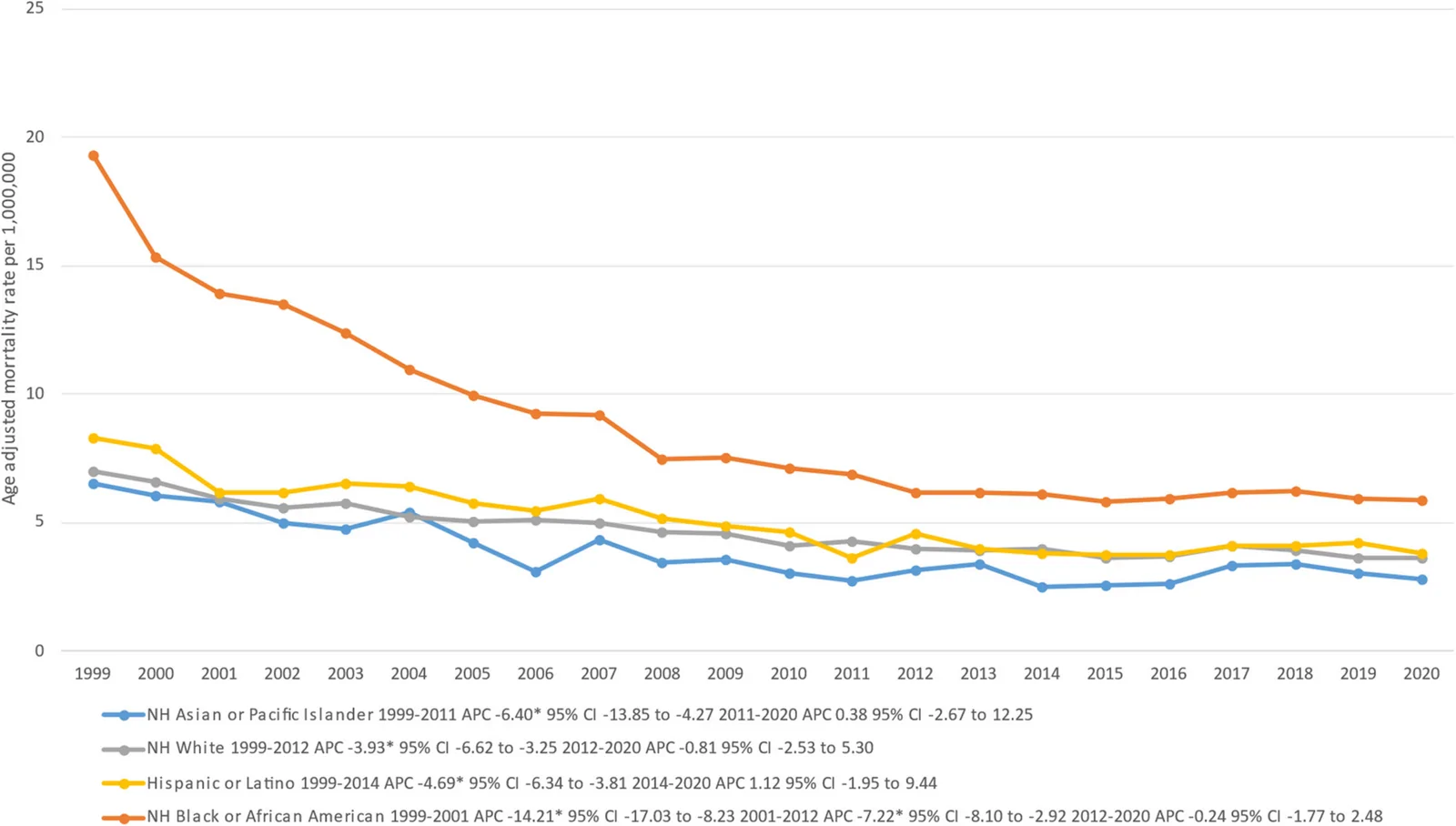
Meningitis-related age-adjusted mortality rates per 1,000,000 population, stratified by race, in the United States, 1999 to 2020. * Annual percentage change is significantly different from zero at α = 0.05. AAMR = age-adjusted mortality rate; APC = annual percentage change; CI = confidence interval

Similarly, Hispanics/Latinos exhibited a significant decline in AAMR from 1999 to 2014 (APC: -4.69*, 95% CI: -6.34 to -3.81) followed by stabilisation for the remainder of the study period. In contrast, NH Asian or Pacific Islanders displayed a distinct trend, with a significant reduction in AAMR from 1999 to 2011 (APC: -6.40*, 95% CI: -13.85 to -4.27) but a slight, nonsignificant increase until 2020 (Supplementary Tables 1, 2 and 4) (Fig. 2).

### Trends by region and state

When the data was stratified in terms of census regions, the AAMR was the highest in the Western region (total: 5.35, 95% CI: 5.24 to 5.47) followed by South (total: 5.33, 95% CI: 5.25 to 5.42), Midwest (total: 4.81, 95% CI: 4.7 to 4.92) and Northeastern region (total: 4.69, 95% CI: 4.57 to 4.81). All regions experienced an initial significant decline in AAMR over different time intervals. The Northeast saw a significant decrease from 1999 to 2013, while the Midwest experienced its decline from 1999 to 2010. In the South, significant reduction occurred between 1999 and 2001, and the West exhibited a notable decrease from 1999 to 2014.

Following their initial significant declines, the Northeast (post-2013) and the Midwest (post-2010) experienced periods of slight decreases in AAMR, extending until 2020. After 2014, the West had a stabilisation period in AAMR, which persisted until the end of the study period.

**Fig. 3:**
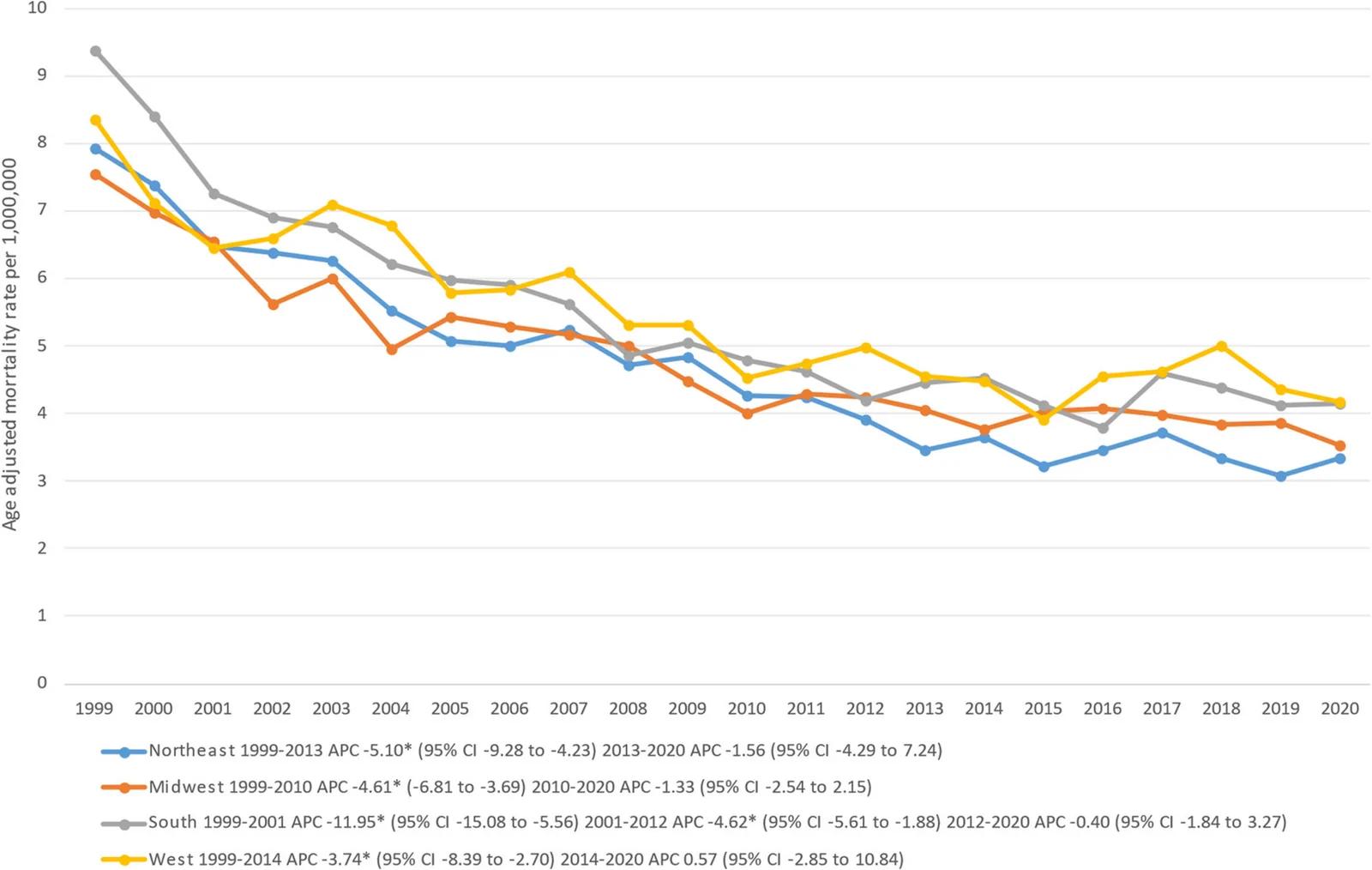
Meningitis-related age-adjusted mortality rates per 1,000,000 population, stratified by Census Regions in the United States, 1999 to 2020. * Annual percentage change is significantly different from zero at α = 0.05. AAMR = age-adjusted mortality rate; APC = annual percentage change; CI = confidence interval

The South differed from other regions, displaying a significant decrease in AAMR from 2001 to 2012 (APC: -4.63*, 95% CI: -5.61 to -1.89), followed by a slight, nonsignificant decline that persisted till 2020 (Supplementary Tables 2 and 5) (Fig. 3).

**Fig. 4:**
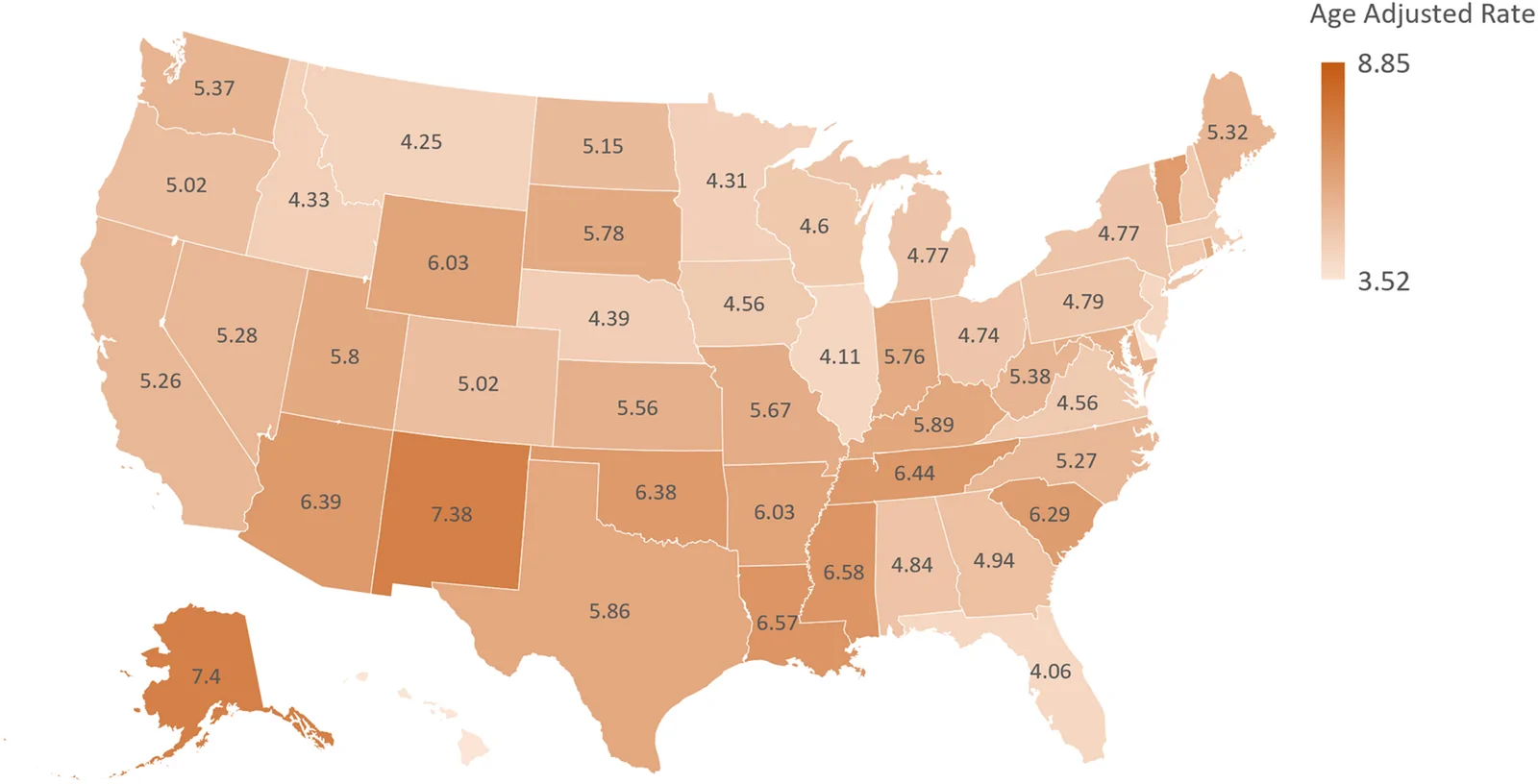
Meningitis-related age-adjusted mortality rates per 1,000,000 population, stratified by states, in the United States, 1999 to 2020. The map depicted in this figure was created by the authors using original analyses and visualisation.

AAMR values varied significantly across states, ranging from 8.85 per 1,000,000 in the District of Columbia to 3.52 per 1,000,000 in Delaware. States in the top 90th percentile, such as the District of Columbia, Alaska, New Mexico, Louisiana, and Mississippi, had AAMRs approximately twice as high as those in the bottom 10th percentile, which included Delaware, Hawaii, New Jersey, Florida, and Illinois (Supplementary Tables 1, 2 and 5) (Fig. 4).

### Trends by urbanisation

**Fig. 5:**
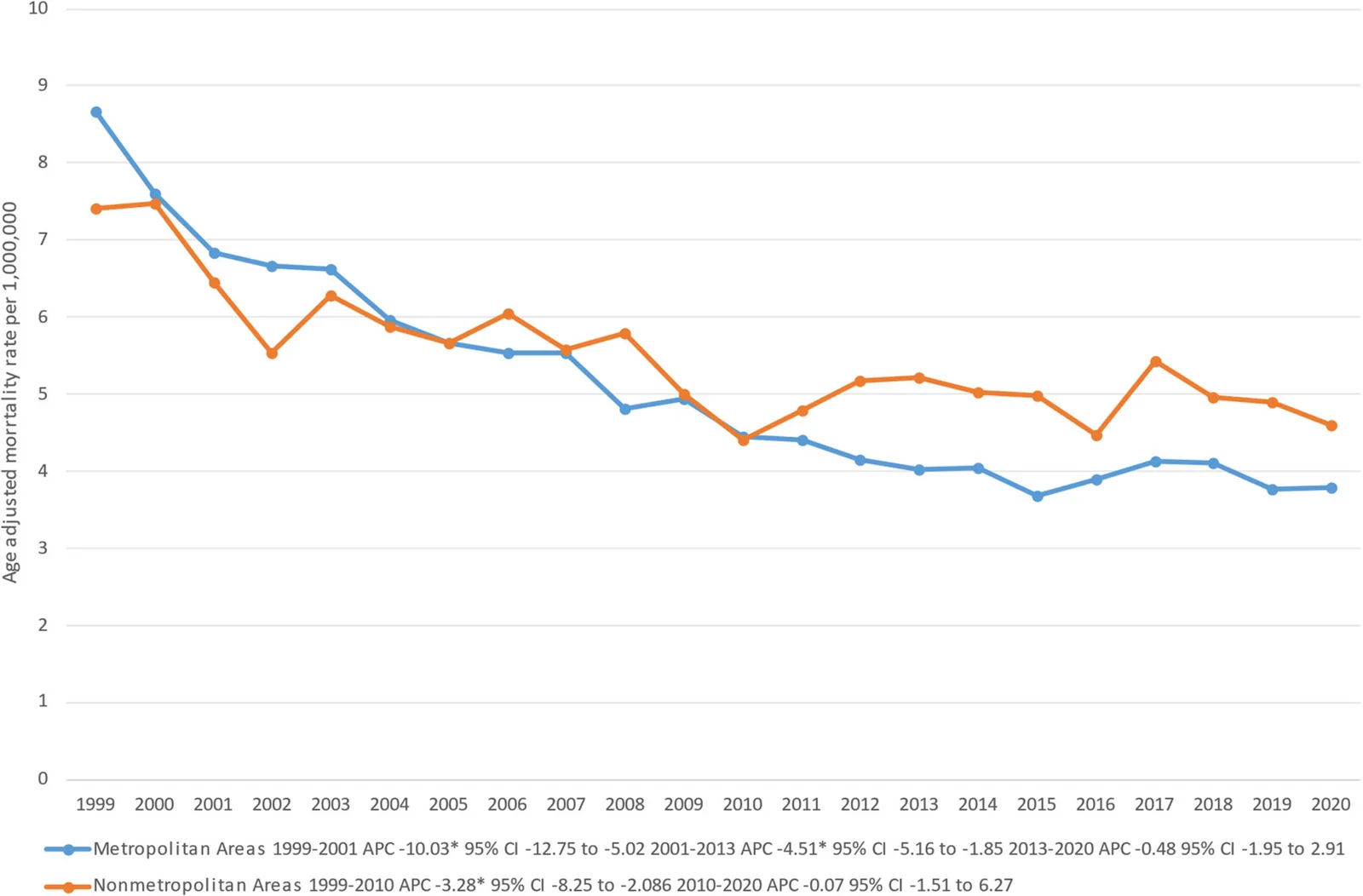
Meningitis-related age-adjusted mortality rates per 1,000,000 population, stratified by urbanisation group, in the United States, 1999 to 2020. * Annual percentage change is significantly different from zero at α = 0.05. AAMR = age-adjusted mortality rate; APC = annual percentage change; CI = confidence interval

Non-metropolitan areas exhibited higher age-adjusted mortality rates. AAMR of non-metropolitan areas dropped significantly from 1999 to 2010 (APC: -3.29*, 95% CI: -8.26 to -2.09), this was followed by a mild drop till 2020 (APC: -0.08, 95% CI: -1.51 to 6.27). Metropolitan areas AAMR followed a significantly decreasing trend from 8.66 in 1999 to 5.66 in 2005 (APC: -10.03*, 95% CI: -12.76 to -5.02). After this, there was another significant downward trend, such that the AAMR reached 4.03 by 2013 (APC: -4.52*, 95% CI: -5.17 to -1.86). The AAMR then started to decrease again slowly till 2020 (APC: -0.49; 95% CI: -1.96 to 2.91) (Supplementary Tables 1 and 6) (Fig. 5).

## Discussion

Through our study investigating the trends pertaining to meningitis-related mortality over the past 2 decades, we report several key findings. To begin with, our analysis highlighted a substantial reduction in meningitis-related mortality over the period spanning 1999–2020.

Across these years, we noticed an initial decade of significant decline, which was followed by a decade of relative stabilisation. This pattern was consistently observed across most of the demographic groups. Moreover, we noticed that disparities along the lines of ethnicity and geography were highly evident, rendering tailored interventions imperative.

### Decline in meningitis-related mortality

Meningitis-related AAMRs decreased from 8.43 per 1,000,000 population in 1999 to 3.88 in 2020, with the steepest reduction occurring in the initial years. Our findings corroborate the existing literature that has highlighted a similar trend [1, 2]. This can be ascribed to the significant advances that have been made in the front of preventive care for meningitis, one of the most monumental of which has been the development of conjugate vaccines for *H. influenzae type b* (PedvaxHIB) in 1987, *S. pneumoniae* (Prevnar-7) in 2000, and *N. meningitidis* (Menactra) in 2005 [3, 4, 7, 8]. A study conducted by Wesserman et al. noted that the introduction of pneumococcal conjugate vaccines, including both PCV-7 and PCV-13, has so far averted more than 282,000 cases of invasive pneumococcal disease (IPD), including 2,780 total deaths [9]. During the late 1990s and early 2000s, universal screening and intrapartum antibiotic prophylaxis for *Group B Streptococcus* (GBS), along with the launch of the Listeria monocytogenes Action Plan by the Food and Drug Administration (FDA) in 2003, have likely contributed to the observed declining trend in neonatal and foodborne infections [10–13].

Another factor behind this change could be the evolution in treatment guidelines. After studies in the Netherlands showed that adjunct dexamethasone significantly reduced mortality from pneumococcal meningitis, the Infectious Diseases Society of America endorsed its use in 2004 [14, 15]. This endorsement likely contributed to a decline in pneumococcal meningitis-related mortality in the U.S. in the following years [1].

### Racial and ethnic disparities

Gross disparities along the lines of racial division were also evident, with NH Black individuals exhibiting an 85% higher AAMR than NH White individuals. Our study expands on the literature that has underlined similar disparities [16]. The divide can be attributed to a host of environmental and social factors, including socioeconomic deprivation, crowded housing, limited access to healthcare, and low parental education, which act synergistically together to place NH Black individuals at a higher risk [17–21]. These circumstances are further exacerbated by the higher prevalence of alcoholism, HIV/AIDS, smoking and diabetes in NH Black individuals, all of which have been linked to a higher burden of meningitis [22–25]. Regardless, assessing inequities within institutions and culturally customised, interdisciplinary interventions may help mitigate the reported racial disparities.

### Sex-based disparities

We also demonstrated persistently higher death rates in men, which is in concordance with a study that found male sex to be an independent predictor of unfavorable outcome in community-acquired bacterial meningitis [26]. A cohort study involving individuals diagnosed with community-acquired meningitis indicated that men are more likely to exhibit concurrent medical problems, such as immunosuppression, HIV/AIDS, and an elevated Charlson Comorbidity Index score [27]. Behavioural factors, such as higher rates of smoking, alcohol consumption, and risky food intake, as well as increased exposure to head trauma, may also contribute to their elevated risk [24, 26, 28]. In addition, the delayed health-seeking behaviour commonly observed in men may have critical consequences in the context of acute diseases such as meningitis, where timely diagnosis and treatment are crucial due to the rapid progression and high mortality risk associated with the condition [29]. Biological factors could also be implicated in higher AAMRs. Studies suggest that females often mount stronger immunological responses to viral meningitis compared to males, likely due to a combination of genetic factors (e.g., X-chromosome-linked immune genes) and hormonal influences (e.g., estrogen’s role in enhancing immune activation) [30, 31]. Moreover, their more favorable response to dexamethasone therapy may also help to explain this difference in outcomes [32].

### Geographic and regional variations

Geographical disparities in mortality range from 3.52 per 1,000,000 population in Delaware to as high as 8.85 per 1,000,000 in the District of Columbia (DC), with the Western and Southern regions experiencing the highest mortality burden. The situation in DC can potentially be explained by a complex interplay of risk factors, including a high population density with a large proportion of vulnerable NH Black individuals, high alcohol consumption, and elevated HIV/AIDS rates [33–36]. On the contrary, Alaska exhibited high AAMRs, despite its low population density, which likely reflects invasive infections from *Haemophilus influenzae serotype a (Hia)* that have predominantly infected Native Alaskan infants in rural communities, threatening to undo the progress made by *Haemophilus influenzae type b* (Hib) vaccination in reducing infant morbidity and mortality [37–39]. 

Although Alaskan Native populations have an extensive tribal healthcare system, remoteness of the populations may still hinder timely intensive care unit admissions [37]. Studies have linked the dry conditions in the meningitis-belt in Africa to higher incidence of meningitis there; this relationship may also hold true for the dry Western and Southern regions of the US [40, 41]. In addition to this, low health literacy, higher HIV/AIDS rates and hospital closures in the South may also contribute to the higher AAMRs [23, 42]. These findings imply the need to conduct large population-based studies in these regions, so that the primary factors behind the observed disparities can be isolated.

### Urban and rural disparities

Another finding we uncovered was that rural areas seemed to fare worse compared to urban ones. The causes behind this may be multifaceted ranging from socioeconomic deprivation, lack of education, inferior infrastructure, and suboptimal vaccination rates, all of which may be inextricably tied to poor outcomes. Studies conducted globally have been observing a correlation between lower social development index (SDI) and higher burden of meningitis, which may be extrapolated plausibly to the rural regions of US as well [43, 44]. Multiple other studies have noted that even marginal delays in treatment of meningitis are associated with significantly worse outcomes, with one such Danish study observing that the risk of death associated with treatment after 2 h was 2.29 (95% CI 1.28–4.09) and increased substantially thereafter [45–47]. Rural residents are prone to facing such critical delays in seeking timely life-saving interventions, owing to poor awareness, subpar infrastructure, and hospital closures [48–50]. Suboptimal vaccination rates may also play a role, with a systematic review highlighting the disparate low meningococcal vaccination rates in rural regions compared to urban ones [51]. Similarly, pneumococcal vaccination rates are also found to be higher in urban residents, and those with higher literacy and internet access [52].

Extending equitable healthcare and telemedicine initiatives to rural areas may mitigate some of the rural-urban disparities in mortality. However, the ultimate solution may lie in policy interventions aimed at ensuring adequate vaccine coverage.

### Future interventions

Reducing meningitis-related deaths requires a multifaceted approach that combines robust vaccination, rapid diagnostics, and equitable healthcare policies. In the short term, efforts should concentrate on achieving immediate impact in high-risk groups and high-burden regions. This includes launching culturally adapted education and free vaccination programs in collaboration with community leaders within non-Hispanic Black communities to address vaccine hesitancy and improve uptake of meningococcal and pneumococcal vaccines. 

Concurrently, establishing telemedicine networks to connect rural clinics in the West and South with tertiary care centers for rapid neurological consultation could significantly reduce critical delays in diagnosis and transfer for patients presenting with meningitis symptoms.

Expansion of vaccine coverage, particularly in underserved and vulnerable population groups, is pivotal in addressing disparate outcomes. Recent advances in the domain of vaccines have sought to broaden the strain coverage of pneumococcal vaccines in order to address the persistent threat of serotype replacement, while the development of combination vaccines also holds the promise to streamline immunization schedules, making it easier to achieve higher vaccination rates [53]. Furthermore, in direct response to the observed mortality plateau, enhancing the granularity of surveillance systems is crucial. The incorporation of whole-genome sequencing (WGS) into routine programs like Active Bacterial Core Surveillance (ABCs) marks a significant advancement, enabling more precise detection of strain variations and antibiotic-resistant clones; leveraging such data to provide real-time regional guidance on empiric therapy could be a powerful tool in high-burden areas [54].

Equally important is the rapid and accurate diagnosis of meningitis. In line with CDC recommendations, healthcare providers are encouraged to maintain a high level of vigilance and recognise even atypical presentations of the disease [55]. Over the medium term, systemic improvements and infrastructure investments are needed to translate this vigilance into action.

Securing funding to equip community hospitals, especially in the Southern and Western regions, with rapid multiplex real-time PCR (RT-PCR) panels would enable swift pathogen identification from a single cerebrospinal fluid sample in about an hour, effectively preventing the critical treatment delays that exacerbate mortality [56]. In the long term, achieving sustainable change will require robust policy interventions and comprehensive prevention strategies. Advocating for federal and state policies that prevent hospital closures in rural areas and expand Medicaid eligibility in non-expanding Southern states is essential for building a resilient healthcare safety net. Complementing this, integrating education on early warning signs of meningitis and the importance of vaccination into school curricula and nationwide community outreach programs, with a specific focus on reaching men and rural populations, can foster long-term improvements in health-seeking behaviour [57]. Ultimately, leveraging the combination of new vaccines, robust surveillance, and equitable access is the path to moving towards the functional elimination of vaccine-preventable bacterial meningitis as a public health problem in the United States.

### Limitations

The limitations of this study include those inherent to the analysis of large administrative datasets and the determination of causes of death. The cross-sectional design and the absence of more granular data inhibited us from performing a thorough multivariable-adjusted analysis to derive causal inferences. The CDC WONDER database lacks individual-level data, including socioeconomic status, key clinical variables such as comorbidity profiles, vaccination history, and duration of symptoms prior to treatment, all of which are critical factors influencing mortality. Furthermore, as with any analysis utilizing death certificate data, there are concerns related to misclassification or inconsistent categorisation of cause of death, for instance, potential misclassification between meningitis and other cerebral inflammatory diseases as the underlying cause of death. Age-specific analyses could not be reliably conducted because CDC WONDER mortality data are aggregated and frequently suppress small death counts in younger age groups, which results in unstable estimates across several strata. Consequently, formal interaction testing between age and other demographic variables was not feasible. Finally, this study did not analyse mortality differences across different meningitis types (bacterial, viral, fungal). Future studies should conduct stratified analyses by etiology to provide evidence for more precise prevention and control strategies.

## Conclusion

Our study provides a comprehensive national analysis of meningitis-related mortality trends in the United States from 1999 to 2020, demonstrating a substantial decline in age-adjusted mortality rates followed by a stabilisation in the past decade. This decline likely reflects advances in public health measures, including widespread vaccination programs, improved prevention strategies, and evolving clinical management of bacterial meningitis. Despite these improvements, significant disparities persist, with higher mortality observed among non-Hispanic Black populations, males, and residents of Western, Southern, and rural regions.

Addressing these disparities will require targeted public health strategies, including improved vaccination coverage, enhanced access to healthcare in underserved communities, and strengthened early diagnostic capacity. Future research incorporating individual-level clinical and socioeconomic data and stratified analyses by meningitis etiology is needed to better understand the drivers of the recent mortality plateau and inform more effective prevention and treatment strategies.

## Supplementary Information

Supplementary Material 1.

## Data Availability

The mortality data analysed in the current study were obtained from the CDC WONDER Multiple Cause of Death, 1999–2020 dataset, which is available at the following URL: https://wonder.cdc.gov/. The data were deposited in the Zenodo database and are available at the following URL: https://doi.org/10.5281/zenodo.20272312.
