# Supplementary material for: Fatal inflammation: understanding meningitis mortality in the United States (1999–2020)
Source: BMC Neurol. 2026 Jun 13;26:525. doi: 10.1186/s12883-026-05060-8 (PMC13488019; doi:10.1186/s12883-026-05060-8)
Supplement: Supplementary file 1 [file 12883_2026_5060_MOESM1_ESM.docx]

**Supplementary Table 1.** Meningitis-related deaths, stratified by sex and race, in the United States, 1999 to 2020.

| **Year** | **Overall** | **Women** | **Men** | **NH White** | **NH Black or African American** | **NH Asian or Pacific Islander** | **Hispanic or Latino** | **Population** |
| --- | --- | --- | --- | --- | --- | --- | --- | --- |
| 1999 | 2324 | 1111 | 1213 | 1477 | 574 | 54 | 200 | 279040168 |
| 2000 | 2112 | 1027 | 1085 | 1406 | 462 | 50 | 179 | 281421906 |
| 2001 | 1925 | 929 | 996 | 1267 | 435 | 53 | 155 | 284968955 |
| 2002 | 1860 | 904 | 956 | 1216 | 415 | 50 | 161 | 287625193 |
| 2003 | 1916 | 925 | 991 | 1265 | 391 | 48 | 196 | 290107933 |
| 2004 | 1759 | 865 | 894 | 1148 | 353 | 58 | 179 | 292805298 |
| 2005 | 1701 | 822 | 879 | 1125 | 329 | 50 | 175 | 295516599 |
| 2006 | 1708 | 844 | 864 | 1155 | 319 | 42 | 176 | 298379912 |
| 2007 | 1718 | 837 | 881 | 1122 | 324 | 56 | 203 | 301231207 |
| 2008 | 1572 | 756 | 816 | 1051 | 265 | 44 | 192 | 304093966 |
| 2009 | 1583 | 735 | 848 | 1055 | 268 | 50 | 192 | 306771529 |
| 2010 | 1449 | 696 | 753 | 958 | 258 | 45 | 170 | 308745538 |
| 2011 | 1479 | 683 | 796 | 1013 | 257 | 40 | 148 | 311591917 |
| 2012 | 1448 | 661 | 787 | 956 | 236 | 51 | 184 | 313914040 |
| 2013 | 1434 | 644 | 790 | 960 | 236 | 55 | 164 | 316128839 |
| 2014 | 1439 | 685 | 754 | 971 | 240 | 44 | 167 | 318857056 |
| 2015 | 1362 | 639 | 723 | 895 | 234 | 44 | 165 | 321418820 |
| 2016 | 1397 | 669 | 728 | 920 | 236 | 49 | 166 | 323127513 |
| 2017 | 1559 | 667 | 892 | 1020 | 257 | 63 | 198 | 325719178 |
| 2018 | 1543 | 681 | 862 | 998 | 256 | 65 | 203 | 327167434 |
| 2019 | 1470 | 665 | 805 | 930 | 254 | 64 | 202 | 328239523 |
| 2020 | 1452 | 673 | 779 | 930 | 255 | 59 | 191 | 329484123 |
| Total | 36210 | 17118 | 19092 | 23838 | 6854 | 1134 | 3966 | 6746356647 |

NH = non-Hispanic.

**Supplementary Table 2**. Annual percentage changes in meningitis-related age-adjusted mortality rates per 1,000,000 in the United States, 1999 to 2020.

| **Year Interval** | **APC (95% CI)** |
| --- | --- |
| **Overall** | |
| 1999-2001 | -9.73*  (-12.36 to -5.02) |
| 2001-2012 | -4.25*  (-4.92 to -2.08) |
| 2012-2020 | -0.87  (-1.97 to 1.57) |
| **Women** | |
| 1999-2012 | -4.92*  (-5.58 to -4.46) |
| 2012-2020 | -1.14  (-2.30 to 0.70) |
| **Men** | |
| 1999-2010 | -4.99*  (-9.02 to -3.87) |
| 2010-2020 | -0.84  (-2.41 to 4.06) |
| **NH Asian or Pacific Islander** | |
| 1999-2011 | -6.40*  (-13.85 to -4.27) |
| 2011-2020 | 0.38  (-2.67 to 12.25) |
| **NH Black or African American** | |
| 1999-2001 | -14.21*  (-17.03 to -8.23) |
| 2001-2012 | -7.22*  (-8.10 to -2.92) |
| 2012-2020 | -0.24  (-1.77 to 2.48) |
| **NH White** | |
| 1999-2012 | -3.93*  (-6.62 to -3.25) |
| 2012-2020 | -0.81  (-2.53 to 5.30) |
| **Hispanic or Latino** | |
| 1999-2014 | -4.69*  (-6.34 to -3.81) |
| 2014-2020 | 1.12  (-1.95 to 9.44) |
| **Northeast Region** | |
| 1999-2013 | -5.10*  (-9.28 to -4.23) |
| 2013-2020 | -1.56  (-4.29 to 7.24) |
| **Midwest Region** | |
| 1999-2010 | -4.61*  (-6.81 to -3.69) |
| 2010-2020 | -1.33  (-2.54 to 2.15) |
| **South Region** | |
| 1999-2001 | -11.95*  (-15.08 to -5.56) |
| 2001-2012 | -4.62*  (-5.61 to -1.88) |
| 2012-2020 | -0.40  (-1.84 to 3.27) |
| **West Region** | |
| 1999-2014 | -3.74*  (-8.39 to -2.70) |
| 2014-2020 | 0.57  (-2.85 to 10.84) |
| **Metropolitan Area** | |
| 1999-2001 | -10.03*  (-12.75 to -5.02) |
| 2001-2013 | -4.51*  (-5.16 to -1.85) |
| 2013-2020 | -0.48  (-1.95 to 2.91) |
| **Nonmetropolitan Areas** | |
| 1999-2010 | -3.28*  (-8.25 to -2.08) |
| 2010-2020 | -0.07  (-1.51 to 6.27) |

APC = annual percentage change; CI = confidence interval; NH = non-Hispanic; * Annual percentage change (APC) is significantly different from zero at α = 0.05.

**Supplementary Table 3**. Overall and sex-stratified meningitis-related age-adjusted mortality rates per 1,000,000 in the United States, 1999 to 2020.

| **Age-Adjusted Mortality Rate (95% CI)** | | | | |
| --- | --- | --- | --- | --- |
| **Year** | **Men** | **Women** | **Overall** | |
| 1999 | 9.59  (9.04 - 10.14) | 7.47  (7.03 - 7.91) | | 8.43  (8.09 - 8.78) |
| 2000 | 8.53  (8.01 - 9.04) | 6.84  (6.42 - 7.26) | | 7.59  (7.27 - 7.91) |
| 2001 | 7.64  (7.16 - 8.12) | 6.09  (5.69 - 6.48) | | 6.78  (6.47 - 7.08) |
| 2002 | 7.2  (6.74 - 7.67) | 5.82  (5.44 - 6.2) | | 6.47  (6.18 - 6.77) |
| 2003 | 7.35  (6.89 - 7.82) | 5.91  (5.53 - 6.3) | | 6.56  (6.26 - 6.85) |
| 2004 | 6.54  (6.1 - 6.97) | 5.44  (5.08 - 5.81) | | 5.93  (5.65 - 6.21) |
| 2005 | 6.28  (5.86 - 6.7) | 5.12  (4.76 - 5.47) | | 5.63  (5.36 - 5.9) |
| 2006 | 6.1  (5.68 - 6.51) | 5.16  (4.81 - 5.51) | | 5.58  (5.31 - 5.84) |
| 2007 | 6.06  (5.66 - 6.47) | 5.13  (4.78 - 5.48) | | 5.54  (5.28 - 5.81) |
| 2008 | 5.48  (5.1 - 5.86) | 4.51  (4.18 - 4.83) | | 4.96  (4.72 - 5.21) |
| 2009 | 5.73  (5.33 - 6.12) | 4.31  (3.99 - 4.62) | | 4.93  (4.69 - 5.18) |
| 2010 | 4.99  (4.62 - 5.35) | 4.02  (3.72 - 4.33) | | 4.45  (4.22 - 4.68) |
| 2011 | 5.2  (4.83 - 5.56) | 3.88  (3.58 - 4.18) | | 4.49  (4.25 - 4.72) |
| 2012 | 5.05  (4.69 - 5.41) | 3.69  (3.4 - 3.98) | | 4.31  (4.08 - 4.54) |
| 2013 | 5.05  (4.69 - 5.41) | 3.51  (3.24 - 3.79) | | 4.22  (4 - 4.44) |
| 2014 | 4.71  (4.37 - 5.05) | 3.79  (3.5 - 4.08) | | 4.18  (3.96 - 4.4) |
| 2015 | 4.38  (4.05 - 4.7) | 3.46  (3.18 - 3.73) | | 3.88  (3.67 - 4.09) |
| 2016 | 4.46  (4.13 - 4.79) | 3.57  (3.29 - 3.85) | | 3.97  (3.75 - 4.18) |
| 2017 | 5.29  (4.94 - 5.65) | 3.45  (3.18 - 3.72) | | 4.31  (4.09 - 4.53) |
| 2018 | 5.05  (4.71 - 5.4) | 3.52  (3.25 - 3.8) | | 4.23  (4.01 - 4.44) |
| 2019 | 4.61  (4.28 - 4.93) | 3.36  (3.1 - 3.63) | | 3.93  (3.72 - 4.14) |
| 2020 | 4.45  (4.13 - 4.77) | 3.38  (3.12 - 3.65) | | 3.88  (3.67 - 4.08) |
| Total | 5.79  (5.71 - 5.87) | 4.53  (4.46 - 4.6) | | 5.1  (5.05 - 5.15) |

CI = confidence interval

**Supplementary Table 4**. Meningitis-related age-adjusted mortality rates per 1,000,000, stratified by race, in the United States, 1999 to 2020

| **Year** | **NH Asian or Pacific Islander** | **NH Black or African American** | **NH White** | **Hispanic or Latino** |
| --- | --- | --- | --- | --- |
| 1999 | 6.49  (4.79 - 8.61) | 19.27  (17.65 - 20.9) | 7  (6.64 - 7.36) | 8.31  (6.98 - 9.65) |
| 2000 | 6.05  (4.41 - 8.09) | 15.31  (13.87 - 16.75) | 6.59  (6.25 - 6.94) | 7.85  (6.52 - 9.18) |
| 2001 | 5.8  (4.26 - 7.71) | 13.9  (12.56 - 15.25) | 5.91  (5.58 - 6.24) | 6.17  (5.05 - 7.3) |
| 2002 | 4.98  (3.62 - 6.68) | 13.5  (12.17 - 14.84) | 5.6  (5.28 - 5.92) | 6.16  (5.09 - 7.22) |
| 2003 | 4.75  (3.45 - 6.38) | 12.39  (11.13 - 13.65) | 5.76  (5.44 - 6.08) | 6.5  (5.46 - 7.54) |
| 2004 | 5.38  (4.03 - 7.04) | 10.96  (9.78 - 12.13) | 5.22  (4.92 - 5.53) | 6.41  (5.36 - 7.47) |
| 2005 | 4.19  (3.08 - 5.57) | 9.92  (8.82 - 11.02) | 5.04  (4.74 - 5.34) | 5.75  (4.79 - 6.72) |
| 2006 | 3.1  (2.2 - 4.24) | 9.24  (8.2 - 10.28) | 5.11  (4.81 - 5.41) | 5.45  (4.54 - 6.37) |
| 2007 | 4.34  (3.25 - 5.67) | 9.19  (8.16 - 10.21) | 4.99  (4.69 - 5.29) | 5.92  (4.99 - 6.85) |
| 2008 | 3.43  (2.46 - 4.65) | 7.44  (6.52 - 8.36) | 4.6  (4.31 - 4.88) | 5.17  (4.35 - 5.99) |
| 2009 | 3.59  (2.65 - 4.76) | 7.51  (6.58 - 8.44) | 4.56  (4.28 - 4.84) | 4.88  (4.11 - 5.64) |
| 2010 | 3.03  (2.19 - 4.1) | 7.14  (6.24 - 8.04) | 4.07  (3.81 - 4.34) | 4.62  (3.84 - 5.39) |
| 2011 | 2.71  (1.92 - 3.72) | 6.87  (6.01 - 7.73) | 4.26  (3.99 - 4.53) | 3.65  (3 - 4.3) |
| 2012 | 3.12  (2.31 - 4.12) | 6.15  (5.34 - 6.95) | 4  (3.73 - 4.26) | 4.57  (3.85 - 5.29) |
| 2013 | 3.38  (2.53 - 4.42) | 6.15  (5.35 - 6.95) | 3.94  (3.68 - 4.2) | 3.97  (3.31 - 4.63) |
| 2014 | 2.52  (1.82 - 3.39) | 6.08  (5.29 - 6.87) | 3.99  (3.73 - 4.25) | 3.81  (3.19 - 4.43) |
| 2015 | 2.55  (1.85 - 3.44) | 5.81  (5.05 - 6.57) | 3.6  (3.35 - 3.85) | 3.72  (3.11 - 4.33) |
| 2016 | 2.61  (1.92 - 3.47) | 5.9  (5.13 - 6.67) | 3.71  (3.46 - 3.96) | 3.74  (3.13 - 4.35) |
| 2017 | 3.35  (2.56 - 4.3) | 6.18  (5.4 - 6.95) | 4.07  (3.8 - 4.33) | 4.11  (3.5 - 4.73) |
| 2018 | 3.38  (2.6 - 4.33) | 6.22  (5.44 - 7) | 3.94  (3.68 - 4.19) | 4.08  (3.49 - 4.67) |
| 2019 | 3.03  (2.33 - 3.89) | 5.91  (5.17 - 6.66) | 3.6  (3.35 - 3.84) | 4.21  (3.6 - 4.82) |
| 2020 | 2.78  (2.1 - 3.6) | 5.85  (5.12 - 6.58) | 3.6  (3.35 - 3.85) | 3.8  (3.23 - 4.36) |
| Total | 3.58  (3.36 - 3.79) | 8.59  (8.38 - 8.79) | 4.64  (4.58 - 4.7) | 4.81  (4.64 - 4.97) |

NH = non-Hispanic; CI = confidence interval.

**Supplementary Table 5.** Meningitis-related age-adjusted mortality rates per 1,000,000, stratified by census region, in the United States, 1999 to 2020

| **Age-Adjusted Mortality Rate 95 % CI** | | | | |
| --- | --- | --- | --- | --- |
| **Year** | **Northeast** | **Midwest** | **South** | **West** |
| 1999 | 7.93  (7.18 - 8.67) | 7.55  (6.87 - 8.22) | 9.36  (8.76 - 9.97) | 8.36  (7.61 - 9.1) |
| 2000 | 7.37  (6.65 - 8.08) | 6.97  (6.32 - 7.61) | 8.39  (7.82 - 8.96) | 7.12  (6.44 - 7.81) |
| 2001 | 6.48  (5.81 - 7.14) | 6.55  (5.93 - 7.17) | 7.26  (6.74 - 7.78) | 6.45  (5.81 - 7.09) |
| 2002 | 6.38  (5.72 - 7.04) | 5.61  (5.03 - 6.18) | 6.91  (6.41 - 7.42) | 6.6  (5.96 - 7.24) |
| 2003 | 6.27  (5.61 - 6.92) | 5.99  (5.4 - 6.58) | 6.77  (6.27 - 7.27) | 7.09  (6.44 - 7.75) |
| 2004 | 5.53  (4.92 - 6.13) | 4.96  (4.43 - 5.5) | 6.22  (5.75 - 6.7) | 6.79  (6.16 - 7.43) |
| 2005 | 5.08  (4.5 - 5.67) | 5.44  (4.88 - 6) | 5.98  (5.52 - 6.44) | 5.78  (5.2 - 6.36) |
| 2006 | 5.01  (4.43 - 5.58) | 5.29  (4.74 - 5.84) | 5.91  (5.46 - 6.36) | 5.83  (5.25 - 6.41) |
| 2007 | 5.23  (4.63 - 5.82) | 5.16  (4.62 - 5.7) | 5.62  (5.18 - 6.06) | 6.1  (5.51 - 6.69) |
| 2008 | 4.71  (4.15 - 5.26) | 4.99  (4.46 - 5.52) | 4.85  (4.45 - 5.25) | 5.31  (4.77 - 5.85) |
| 2009 | 4.84  (4.27 - 5.41) | 4.47  (3.98 - 4.97) | 5.05  (4.64 - 5.46) | 5.3  (4.77 - 5.84) |
| 2010 | 4.26  (3.72 - 4.79) | 4.01  (3.54 - 4.48) | 4.79  (4.39 - 5.18) | 4.52  (4.03 - 5.01) |
| 2011 | 4.23  (3.7 - 4.75) | 4.29  (3.8 - 4.77) | 4.61  (4.22 - 4.99) | 4.73  (4.23 - 5.23) |
| 2012 | 3.9  (3.39 - 4.4) | 4.24  (3.76 - 4.72) | 4.19  (3.83 - 4.56) | 4.98  (4.47 - 5.49) |
| 2013 | 3.45  (2.99 - 3.92) | 4.04  (3.57 - 4.5) | 4.46  (4.09 - 4.83) | 4.55  (4.07 - 5.04) |
| 2014 | 3.64  (3.15 - 4.12) | 3.76  (3.3 - 4.21) | 4.52  (4.15 - 4.9) | 4.47  (4 - 4.94) |
| 2015 | 3.21  (2.75 - 3.66) | 4.02  (3.55 - 4.48) | 4.12  (3.77 - 4.48) | 3.9  (3.47 - 4.34) |
| 2016 | 3.46  (2.99 - 3.93) | 4.08  (3.6 - 4.55) | 3.79  (3.45 - 4.13) | 4.56  (4.09 - 5.03) |
| 2017 | 3.72  (3.24 - 4.21) | 3.99  (3.53 - 4.45) | 4.6  (4.23 - 4.97) | 4.61  (4.14 - 5.08) |
| 2018 | 3.33  (2.88 - 3.78) | 3.83  (3.38 - 4.28) | 4.38  (4.02 - 4.74) | 5  (4.52 - 5.48) |
| 2019 | 3.07  (2.63 - 3.51) | 3.87  (3.42 - 4.32) | 4.12  (3.77 - 4.46) | 4.36  (3.91 - 4.81) |
| 2020 | 3.35  (2.9 - 3.81) | 3.53  (3.1 - 3.96) | 4.15  (3.8 - 4.49) | 4.16  (3.73 - 4.6) |
| Total | 4.69  (4.57 - 4.81) | 4.81  (4.7 - 4.92) | 5.33  (5.25 - 5.42) | 5.35  (5.24 - 5.47) |

CI = confidence interval

**Supplementary Table 6.** Meningitis-related age-adjusted mortality rates per 1,000,000, stratified by states, in the United States, 1999 to 2020

| **State** | **Age-Adjusted Mortality Rate**  **(95% CI)** |
| --- | --- |
| Alabama | 4.84  (4.42 - 5.25) |
| Alaska | 7.4  (5.92 - 8.88) |
| Arizona | 6.39  (5.98 - 6.81) |
| Arkansas | 6.03  (5.44 - 6.62) |
| California | 5.26  (5.1 - 5.42) |
| Colorado | 5.02  (4.59 - 5.44) |
| Connecticut | 4.62  (4.16 - 5.08) |
| Delaware | 3.52  (2.77 - 4.42) |
| District of Columbia | 8.85  (7.22 - 10.48) |
| Florida | 4.06  (3.87 - 4.25) |
| Georgia | 4.94  (4.64 - 5.25) |
| Hawaii | 3.53  (2.88 - 4.19) |
| Idaho | 4.33  (3.63 - 5.02) |
| Illinois | 4.11  (3.88 - 4.35) |
| Indiana | 5.76  (5.37 - 6.15) |
| Iowa | 4.56  (4.07 - 5.06) |
| Kansas | 5.56  (4.98 - 6.14) |
| Kentucky | 5.89  (5.41 - 6.36) |
| Louisiana | 6.57  (6.07 - 7.07) |
| Maine | 5.32  (4.53 - 6.11) |
| Maryland | 5.57  (5.16 - 5.98) |
| Massachusetts | 4.5  (4.17 - 4.83) |
| Michigan | 4.77  (4.49 - 5.06) |
| Minnesota | 4.31  (3.94 - 4.67) |
| Mississippi | 6.58  (5.96 - 7.2) |
| Missouri | 5.67  (5.28 - 6.07) |
| Montana | 4.25  (3.42 - 5.09) |
| Nebraska | 4.39  (3.75 - 5.02) |
| Nevada | 5.28  (4.68 - 5.87) |
| New Hampshire | 4.57  (3.82 - 5.32) |
| New Jersey | 4.09  (3.81 - 4.37) |
| New Mexico | 7.38  (6.58 - 8.17) |
| New York | 4.77  (4.57 - 4.98) |
| North Carolina | 5.27  (4.96 - 5.58) |
| North Dakota | 5.15  (4.1 - 6.39) |
| Ohio | 4.74  (4.48 - 5) |
| Oklahoma | 6.38  (5.84 - 6.92) |
| Oregon | 5.02  (4.56 - 5.48) |
| Pennsylvania | 4.79  (4.55 - 5.04) |
| Rhode Island | 5.7  (4.78 - 6.62) |
| South Carolina | 6.29  (5.81 - 6.77) |
| South Dakota | 5.78  (4.7 - 6.86) |
| Tennessee | 6.44  (6.02 - 6.85) |
| Texas | 5.86  (5.65 - 6.07) |
| Utah | 5.8  (5.13 - 6.47) |
| Vermont | 6.33  (5.11 - 7.75) |
| Virginia | 4.56  (4.24 - 4.87) |
| Washington | 5.37  (5 - 5.74) |
| West Virginia | 5.38  (4.69 - 6.08) |
| Wisconsin | 4.6  (4.23 - 4.97) |
| Wyoming | 6.03  (4.74 - 7.57) |
